# RNA Polymerase III Subunit POLR3G Regulates Specific Subsets of PolyA^+^ and SmallRNA Transcriptomes and Splicing in Human Pluripotent Stem Cells

**DOI:** 10.1016/j.stemcr.2017.04.016

**Published:** 2017-05-09

**Authors:** Riikka J. Lund, Nelly Rahkonen, Maia Malonzo, Leni Kauko, Maheswara Reddy Emani, Virpi Kivinen, Elisa Närvä, Esko Kemppainen, Asta Laiho, Heli Skottman, Outi Hovatta, Omid Rasool, Matti Nykter, Harri Lähdesmäki, Riitta Lahesmaa

**Affiliations:** 1 Turku Centre for Biotechnology, University of Turku and Åbo Akademi University, Turku 20520, Finland; 2 Department of Computer Science, Aalto University, Espoo 02150, Finland; 3 Faculty of Medicine and Life Sciences, BioMediTech, University of Tampere, Tampere 33014, Finland; 4 Department CLINTEC, Karolinska Institutet, Karolinska University Hospital Huddinge, Stockholm 171 77, Sweden

**Keywords:** pluripotency, human embryonic stem cell, polymerase III, transcriptome, smallRNA, polyA^+^ RNA, splicing, mitochondria

## Abstract

POLR3G is expressed at high levels in human pluripotent stem cells (hPSCs) and is required for maintenance of stem cell state through mechanisms not known in detail. To explore how POLR3G regulates stem cell state, we carried out deep-sequencing analysis of polyA^+^ and smallRNA transcriptomes present in hPSCs and regulated in POLR3G-dependent manner. Our data reveal that POLR3G regulates a specific subset of the hPSC transcriptome, including multiple transcript types, such as protein-coding genes, long intervening non-coding RNAs, microRNAs and small nucleolar RNAs, and affects RNA splicing. The primary function of POLR3G is in the maintenance rather than repression of transcription. The majority of POLR3G polyA^+^ transcriptome is regulated during differentiation, and the key pluripotency factors bind to the promoters of at least 30% of the POLR3G-regulated transcripts. Among the direct targets of POLR3G, *POLG* is potentially important in sustaining stem cell status in a POLR3G-dependent manner.

## Highlights

• A specific subset of transcriptome is expressed in POLR3G-dependent manner in hPSCs
• The primary function of POLR3G is in the maintenance of transcription
• mtDNA polymerase gamma (POLG) is among the direct targets of POLR3G

## Teaser

In this article, Lund and colleagues show that RNA polymerase III subunit G (POLR3G) regulates a specific subset of transcriptome in human pluripotent stem cells. The primary function of POLR3G is in the maintenance of transcription. Among the direct targets of POLR3G is mtDNA polymerase γ, with potential importance in maintenance of stem cell state in a POLR3G-dependent manner.

## Introduction

Human embryonic stem cells (hESCs) are pluripotent cells having a unique capacity to self-renew and differentiate into all specialized cell types found in somatic tissues (Thomson et al., 1998). Similar properties are gained by human induced pluripotent stem cells (hiPSCs) reprogrammed from cells found in adult tissues (Yu et al., 2007, Takahashi et al., 2007). The core factors known to be crucial for maintenance and control of pluripotency include POU5F1, SOX2, and NANOG (Boyer et al., 2005, Chambers and Tomlinson, 2009). These transcription factors operate in activation or repression of genes important for pluripotency and differentiation, and form an autoregulatory loop to positively regulate their own expression. In addition, POU5F1 and SOX2, in combination with either KLF4 and CMYC or NANOG and LIN28, were used in the first studies reprogramming adult cells back to the pluripotent state, highlighting the importance of these factors in the regulation of pluripotency (Yu et al., 2007, Takahashi et al., 2007). Moreover, epigenetic regulation and post-transcriptional mechanisms, such as microRNAs (miRNAs), are important in the regulation of pluripotency (Young, 2011). However, the mechanisms underlying the unique property of pluripotency are still not completely understood.

In eukaryotic cells, three DNA-directed RNA polymerases, polymerases I, II, and III (Pol I–III), regulate transcription of different sets of target genes. Pol III regulates transcription of structural RNAs (tRNAs and 5S rRNAs) and several small non-protein-coding RNAs (ncRNAs) including miRNAs (Ozsolak et al., 2008, Borchert et al., 2006, Oler et al., 2010, Dieci et al., 2007). Pol III, like Pol I and Pol II, is composed of multiple subunits of which POLR3G (DNA-directed RNA polymerase III subunit, RPC32, RPC7) is a Pol III-specific subunit with no counterpart in Pol I or Pol II. POLR3G subunit is needed for transcriptional initiation of Pol III, thus being important for the proper function of this polymerase (Wang and Roeder, 1997).

Intriguingly, consistent with previous studies (Wong et al., 2011, Enver et al., 2005, Haurie et al., 2010), our data show that POLR3G is highly expressed in pluripotent stem cells and is required for maintenance of the pluripotent state. However, the mechanisms by which how POLR3G contributes to maintenance of stem cell state and its targets in stem cells are not well known. To explore the function of POLR3G in stem cells, we have carried out deep transcriptome analysis of mRNAs and smallRNAs present in hESCs and regulated in a POLR3G-dependent manner. Our data reveal a specialized function for POLR3G in the transcriptional maintenance of the hESC state and regulation of developmental programs through a specific subset of coding and non-coding transcriptomes and regulation of alternative splicing.

## Results

### Deep PolyA^+^ Transcriptome of hESCs

**Figure 1:**
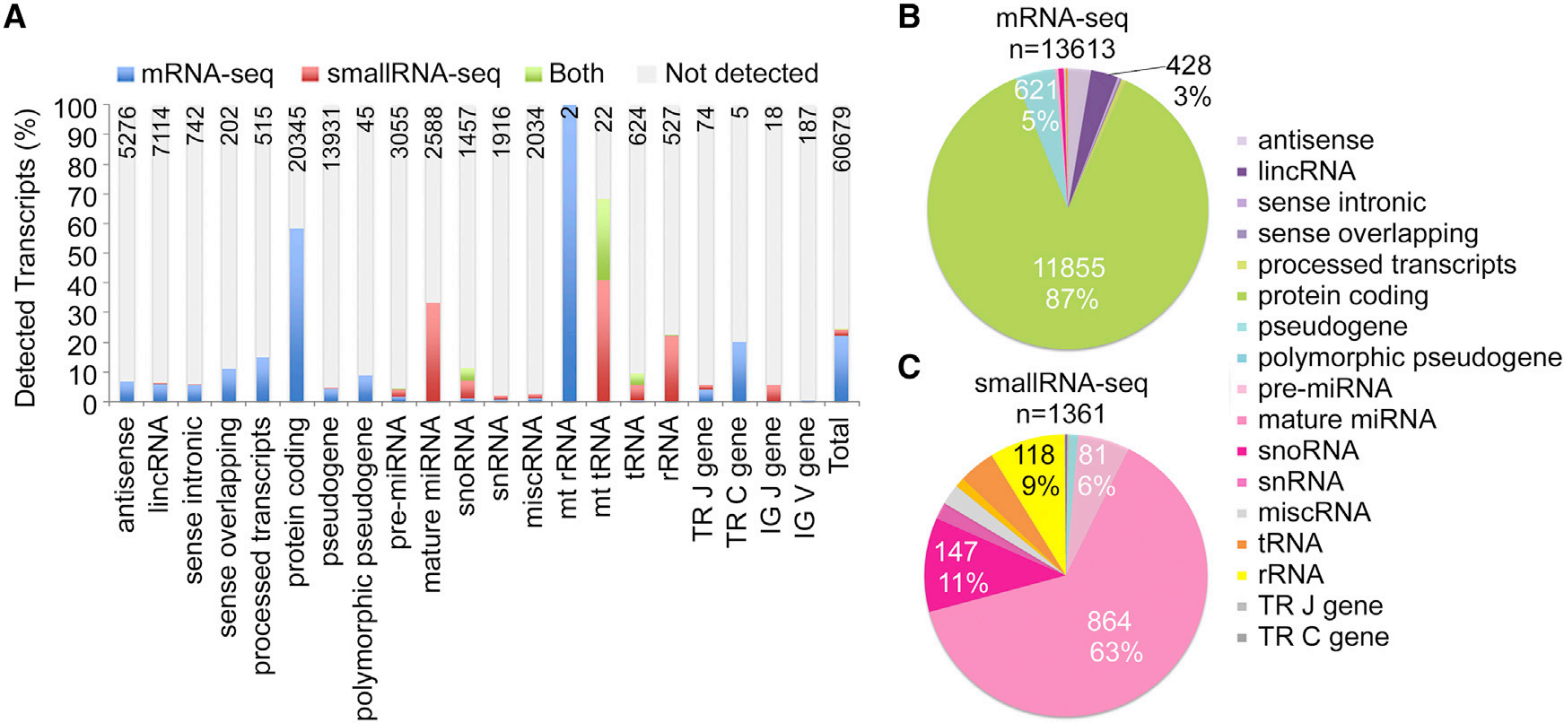
Deep SmallRNA and PolyA^+^ RNA Reference Transcriptome of Human Embryonic Stem Cells (A–C) PolyA^+^ and smallRNAs were isolated from three independent experiments with two different human embryonic stem cells lines (H9p38, HS360p62, HS360p66). The deep transcriptome was analyzed with smallRNA-seq and mRNA-seq. The sequence data were mapped to the hg19 reference genome assembly and miRBase database (based on hg19 build). Transcriptome annotations were taken from gencode v19 and tRNA annotations from UCSC genome browser (in addition to miRBase). The polyA^+^ transcripts with a minimum RPKM value of 0.5 and the smallRNA transcripts with a minimum RPM value of 0.5 were extracted from the data and classified into different transcript type categories. In (A) the bars represent the proportion of each transcript class detected in hESCs in comparison with the total number (indicated in the figure) of each transcript type present in the reference genome. The proportions of different transcript classes present in hESCs and detected with mRNA-seq (B) and smallRNA-seq (C) are visualized as pie charts.

To explore the regulation of transcription by POLR3G in hESCs, we first established a comprehensive reference transcriptome for hESCs. For this purpose we explored how large a proportion of the different transcript types are present in hESCs. Using mRNA sequencing (mRNA-seq), from the total number of 181–224 × 10^6^ reads per sample we detected altogether 13,613 polyA^+^ transcripts, which were present in all of the three hESC samples examined with a minimum RPKM (reads per kilobase per million mapped reads) value of 0.5 (Table S1A). This represents 22% of the total 60,679 transcripts present in the hg19 and miRBase reference genomes (Figure 1A). The most abundant class of transcripts detected in hESCs was protein-coding genes, which included 11,855 transcripts representing 58% of the protein-coding genes in the gencode (hg19) annotations (Figure 1A) and 87% of all the polyA^+^ transcripts that were detected in hESC transcriptome (Figure 1B). The three other most abundant classes of transcripts were pseudogenes (n = 621, 5% of hESC transcriptome), long intervening non-coding RNAs (lincRNAs) (n = 428, 3% of hESC transcriptome), and antisense transcripts (n = 361, 3% of hESC transcriptome). However, in all these other classes less than 5% of the transcripts present in the reference human genome were detected with mRNA-seq (Figure 1A).

### SmallRNA Transcriptome of hESCs

With smallRNA-seq we detected altogether 1,361 transcripts present in all hESC samples with a minimum read per kilobase of 0.5 and a maximum length of 250 bp (Table S1B). This represented 2% of all the transcripts in hg19 and miRBase reference genomes (Figure 1A). The most abundant class of smallRNA transcripts was mature miRNAs (n = 864), which represent 33% of the miRNAs in the miRBase reference annotations or 63% of all the smallRNAs that were detected in hESC small transcriptome (Figure 1C). The other most abundant classes of transcripts were small nucleolar RNAs (snoRNAs) (n = 147 transcripts, 11% of hESC smallRNA transcriptome) and pre-miRNAs (n = 81, 6% hESC small transcriptome), rRNAs (n = 118, 9% of hESC smallRNA transcriptome), and tRNAs (n = 57, 4% of hESC smallRNA transcriptome).

### POLR3G-Dependent PolyA^+^ Transcriptome in hESCs

**Figure 2:**
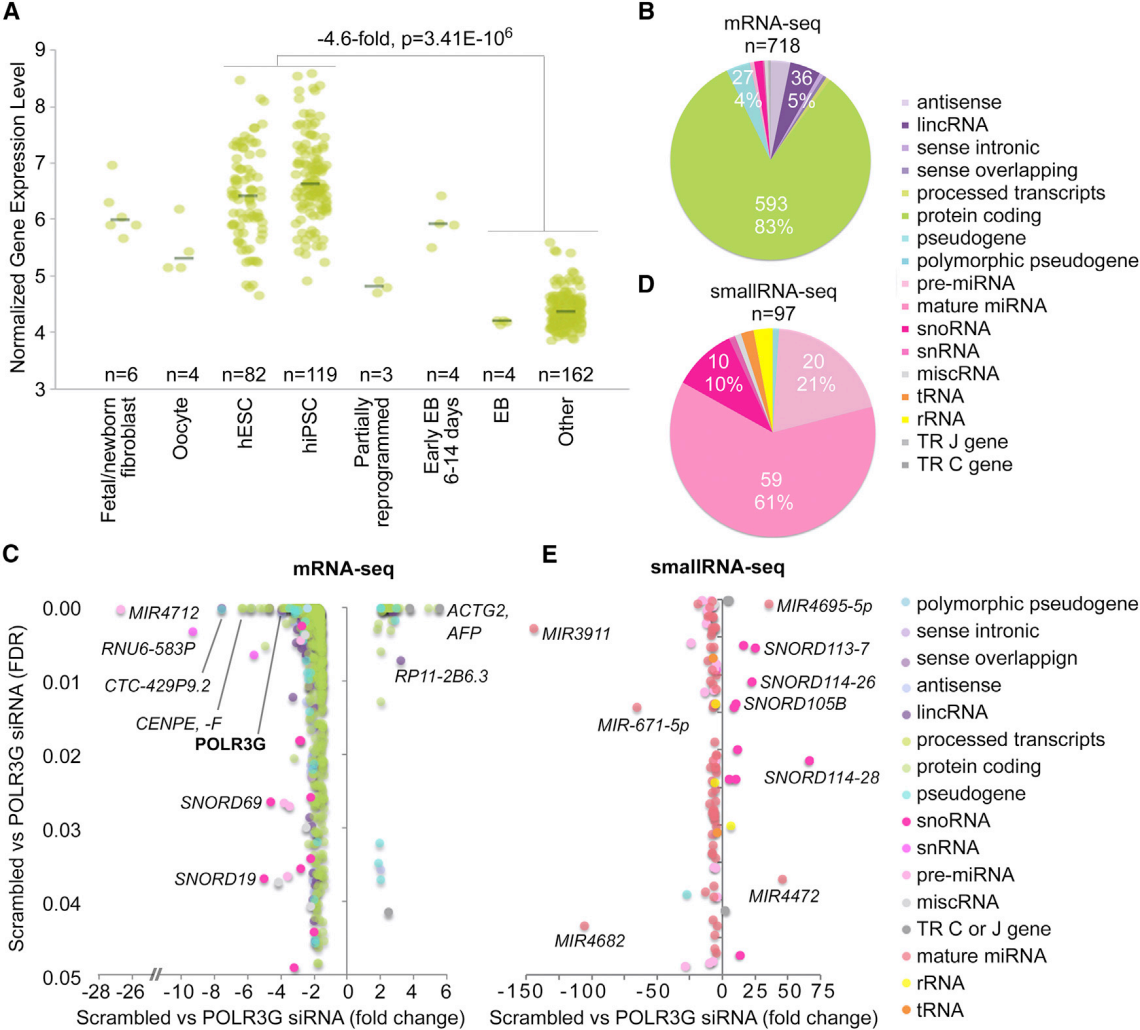
POLR3G-Dependent Human Embryonic Stem Cell Transcriptome (A) Normalized gene expression levels for POLR3G from ESTOOLS data@hand database as measured with Affymetrix arrays in 384 samples. The combined number (n) of replicated samples from different studies is indicated in the figure. Average fold changes and statistical significance (unpaired t test) between hPSCs in comparison with late embryonic bodies (EB) and other cell types is indicated in the figure. See also Figure S1. (B) The proportions of different polyA^+^ transcript types regulated in POLR3G-dependent manner in hESCs with minimum fold change of 1.5 and FDR ≤0.05 in three independent biological replicates. (C) The fold changes and FDR values for all POLR3G-dependent transcripts in mRNA-seq data. (D) The proportions of different smallRNA transcript types regulated in POLR3G-dependent manner in hESCs with minimum fold change of 1.5 and FDR ≤0.05 in four siRNA samples in comparison with three non-targeted siRNA controls. (E) The average fold changes and FDR values for all POLR3G-dependent transcripts with size below 250 bp in smallRNA-seq data from four siRNA samples in comparison with three non-targeted siRNA controls. See also Figure S2.

Consistent with previous findings (Enver et al., 2005, Wong et al., 2011, Haurie et al., 2010), we found both POLR3G mRNA and protein to be expressed at high level in hPSCs and to be rapidly downregulated during early differentiation (Figures 2A, S1A, and S1B). Expression of POLR3G protein was reciprocal to alternative form POLR3GL, which was induced in response to differentiation (Figures S1A and S1B). In addition, POLR3G was expressed consistently in a POU5F1-dependent manner (Figure S1C). To study the function and importance of POLR3G in hESCs, we carried out small interfering RNA (siRNA)-mediated knockdown of POLR3G in two different hESC lines (H9 and HS360) with two different siRNAs and siRNA pool (see Supplemental Experimental Procedures). Silencing of POLR3G led to clear loss of morphology typical for pluripotent hESCs and decreased the number of cells present in the cultures (Figure S2A). In particular with siRNA1, due to rapid and strong decrease in cell numbers, not enough material was always obtained for further analysis, indicating the importance of POLR3G in hESC maintenance. We did not observe an increase in the number of dead cells in response to POLR3G knockdown, although these cells would have been washed off in the daily media change (Figure S2A).

We next examined the function of POLR3G in the regulation of hESC transcriptome. Comparison of the polyA^+^ transcriptomes of hESCs before and after POLR3G silencing revealed changes in expression of 718 transcripts representing 5% of the hESC transcriptome (Table S2A). The most abundant transcriptional class regulated by POLR3G (Figure 2B) was protein-coding genes (n = 593), which represented 83% of all the polyA^+^ transcripts regulated by POLR3G and 5% of the protein-coding genes present in hESC transcriptome. The other less abundant polyA^+^ transcript classes regulated by POLR3G included 36 lincRNAs (5%), 27 pseudogenes (4%), and 23 antisense transcripts (6% of POLR3G-dependent transcripts). No increase in the expression of alternative variant POLR3GL was detected at either mRNA or protein level (data not shown), although this variant is induced during early differentiation and is expressed in reciprocal manner to POLR3G (Figure S1). Consistently with our observations from cell cultures and cell counts (Figure S2), among the genes with the strongest decrease in response to POLR3G was a marker of proliferation, MKI67 (fold change = −5.9, false discovery rate [FDR] = 1.71 × 10^−8^, Table S2), indicating decreased proliferation of the cells. Our findings show that POLR3G regulates a specific subset of transcripts in hESCs consisting of multiple transcript types. However, the most abundant POLR3G-dependent transcript group in our mRNA-seq data is clearly the protein-coding genes. Most of the POLR3G-dependent polyA^+^ transcripts were decreased (n = 681, 94.8%) rather than increased (n = 37, 5.2%) in response to POLR3G knockdown (Figure 2C), indicating that POLR3G has a function primarily in maintenance rather than repression of transcription in hESCs.

### POLR3G-Dependent SmallRNA Transcriptome in hESCs

The smallRNA-seq revealed changes in 97 POLR3G-dependent smallRNAs representing 7% of the hESC smallRNA transcriptome (Table S2B). The most abundant transcriptional class regulated by POLR3G (Figure 2D) was mature miRNAs (n = 59) representing 61% of all the smallRNAs regulated by POLR3G and 7% of the miRNAs present in hESC smallRNA transcriptome. The other abundant smallRNA classes regulated by POLR3G included pre-miRNAs (n = 20) and snoRNAs (n = 10), representing 21% and 10% of all POLR3G-dependent smallRNAs, respectively. Similarly to polyA^+^ transcriptome, most of the POLR3G-dependent smallRNAs (n = 84, 87%) were downregulated and only a small proportion (n = 13, 13%) was upregulated in response to POLR3G knockdown (Figure 2E and Table S2B). Interestingly, most of the smallRNAs (n = 10, 77%) induced in response to POLR3G silencing were snoRNAs. Half of these were from imprinted locus in the chromosome 14q32. In comparison with polyA^+^ transcripts, the impact of POLR3G depletion on smallRNAs was strong in magnitude. For polyA^+^ transcripts the average fold change for repressed transcripts in response to POLR3G silencing was 1.9, whereas for smallRNAs it was 5.9. These results indicate that in addition to multiple types of polyA^+^ transcripts, POLR3G is required for maintenance of a specific subset of miRNAs in hESCs. In addition, POLR3G represses transcription of a small fraction of snoRNAs.

### Functional Enrichment Analysis of POLR3G-Regulated Transcripts

To examine the function of the POLR3G-dependent transcripts, we performed pathway analysis with the Ingenuity Pathway Analysis Tool (Qiagen). Consistent with our findings, the results linked the POLR3G-regulated polyA^+^ transcripts to important functions in the cellular maintenance and regulation of early developmental programs (Table 1). The functions of POLR3G-dependent non-coding transcripts, including smallRNAs, are largely unknown.

A total of 114 molecules linked to pluripotency function were found in the Ingenuity Pathway database. Of these, eight (*DNMT1*, *APC*, *SMARCA4*, *NIPBL*, *RIF1*, *RTF1*, *TET1*, and *LIN28A*) showed downregulation from −1.5-fold to −2.9-fold (FDR < 0.05) in response to POLR3G knockdown. In addition, several of the miRNAs predicted to target these factors were regulated in a POLR3G-dependent manner. However, most of them were downregulated rather than upregulated. Only two of the miRNAs were upregulated (*hsa-miR-4472*, 45.89-fold, FDR = 0.04; and *hsa-miR-4695-5p*, 24.63-fold, FDR = 0.0005) and their predicted targets *RTF1* (−1.62, FDR = 0.0003) and *RIF1* (−1.55, FDR = 0.004), respectively, were downregulated. Among the downregulated pluripotency-associated genes was also *LIN28A* (−1.88, FDR = 2.12 × 10^−4^), which has a well-known function in the regulation of mouse ESC self-renewal through suppression of *let-7* miRNA maturation (Shyh-Chang and Daley, 2013). *Let-7* miRNAs again repress *LIN28A* during stem cell differentiation. Therefore, we looked into the *let-7* levels in the data and found increased levels of *hsa-let-7b-5p* (4.35-fold, p = 0.02) and *hsa-let-7c* (5.21-fold, p = 0.01) with unadjusted p values in response to POLR3G silencing. No consistent changes were observed for the other *let-7* family members.

Taken together, based on functional enrichment analysis, POLR3G is required for maintenance of transcripts important in regulation of cellular maintenance, proliferation, and early developmental programs. Importantly, the functions of the POLR3G-dependent non-coding RNAs are largely unknown, highlighting the lack of knowledge in the importance of non-coding transcriptome in the regulation of pluripotency.

### Regulation of POLR3G-Dependent Transcripts during Early Differentiation

To study whether the POLR3G-dependent transcripts are regulated during differentiation of hESCs, we examined regulation of these transcripts in the deeply sequenced RNA-seq data available on differentiation of hESCs into three different germ cell lineages (endoderm, mesoderm, and ectoderm) by Gifford et al. (2013). Of our 718 POLR3G-regulated polyA^+^ transcripts, 537 were present in the data by Gifford et al. (2013). Of these, 70% (n = 377) showed at least 1.5-fold change (p value cutoff 0.05) in response to differentiation (Table S3). This represented 3.8% of the all 10,010 differentially regulated genes in the data by Gifford et al. (2013). The POLR3G-regulated transcripts included both induced and repressed genes and were associated with all three germ cell lineages endoderm (98 down, 120 up), ectoderm (108 down, 116 up), and mesoderm (151 down, 104 up). From these observations we can conclude that POLR3G regulates a specific subset of transcripts in hESCs, most of which are differentially regulated during early differentiation of hESCs to all the different germ cell lineages.

### Impact of POLR3G Silencing on Alternative Splicing of Transcripts

We next analyzed the impact of POLR3G silencing on the alternative splicing of transcripts. Using the rMATS method (replicate multivariate analysis of transcript splicing) (Shen et al., 2014), the number of splicing events detected in the samples ranged from thousands (retained intron) to tens of thousands (skipped exon) based on counts from reads mapping to exon junctions and alternative exons (and similar numbers based on just junction counts), whereas the combined number of alternative splicing events (ASEs) from both junction counts and counts from junction and alternative exon reads (FDR < 0.05, ΔΨ > 0.2) for the following subclasses were: 109 (71) skipped exon (SE), 25 (16) alternative 5′ splice site (A5SS), 15 (7) alternative 3′ splice site (A3SS), 62 (49) retained intron (RI), and 22 (11) mutually exclusive exons (MXE) (Table S4). The numbers in parentheses indicate the number of ASEs wherein the sign of ΔΨ was consistent among all three case-control pairs (in contrast to ASEs that had too few reads in some samples or those wherein the sign of ΔΨ varied among case-control pairs).

Splicing analysis with MISO (mixture of isoforms) (Katz et al., 2010) was performed independently for each of the three case-control pair replicates and splicing events meeting the filtering criteria (Bayes factor ≥5, ΔΨ >0.2, number of reads supporting inclusion/exclusion isoform for case/control samples, or vice versa, ≥10) for all the three replicate pairs and with consistent sign of ΔΨ were considered significant. Using these ad hoc filtering criteria, only six ASEs were detected (Table 2).

Given the relatively small number of statistically significant splicing events from MISO analysis and discrepancies in the splicing event annotations, only one ASE, skipped exon of *HDAC7* gene (exon 9 of RefSeq NM_015401), was detected by both rMATS (FDR = 8.82 × 10^−22^, ΔΨ = 0.29) and MISO (Bayes factor = 1.00E+12, ΔΨ = 0.21–0.37) (Figure S3). This ASE was subjected to further experimental validation with qRT-PCR, which confirmed increased expression (fold change 1.52–7.03, p = 0.09) of the variant lacking the exon after silencing of POLR3G in all four biological replicates examined (data not shown).

### Enrichment of Pluripotency Factors in the Proximity of POLR3G-Dependent Transcripts

**Figure 3:**
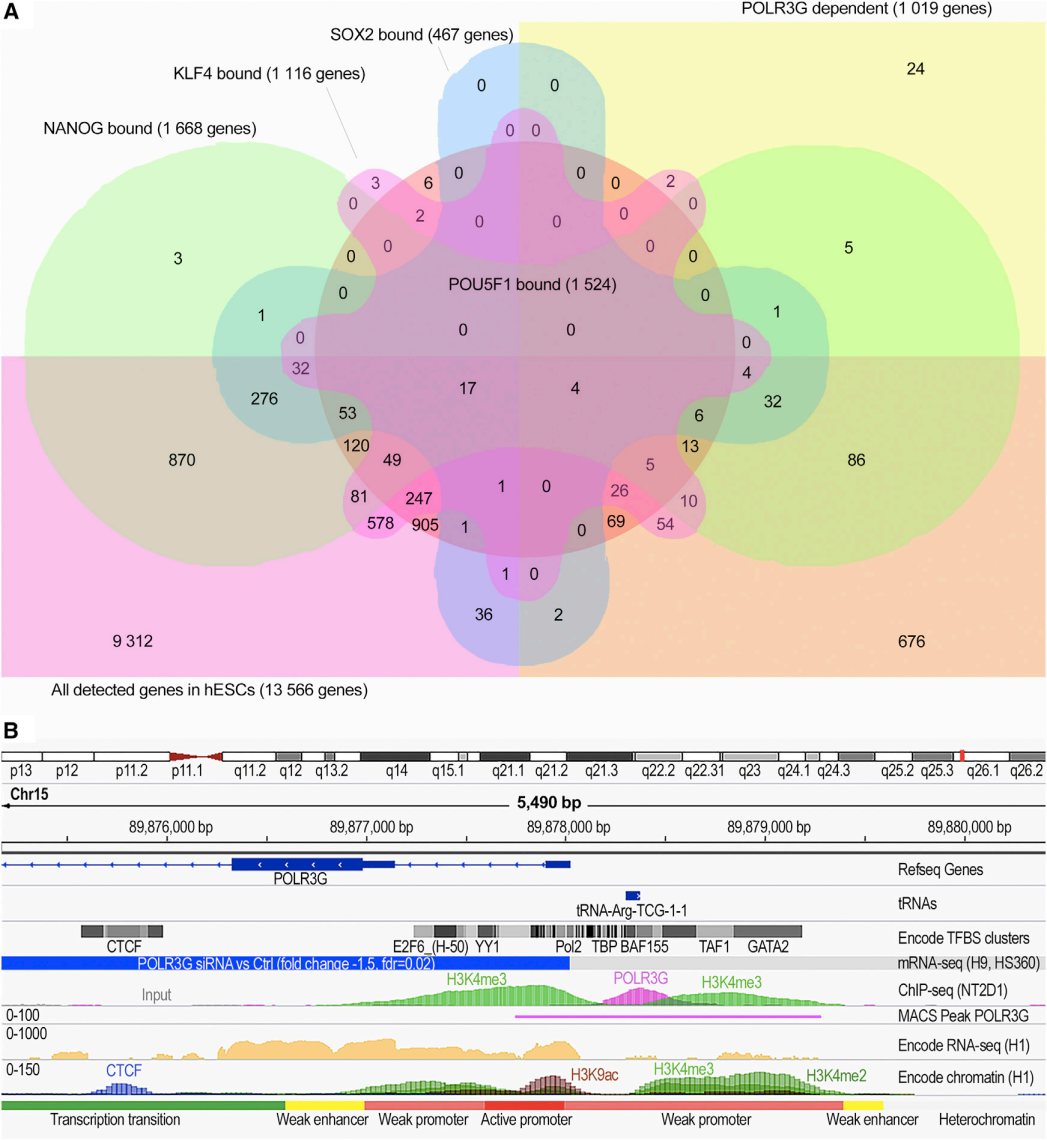
Overlap of the Genomic Binding Sites of Known Pluripotency Regulators and POLR3G with the POLR3G-Regulated Transcriptome (A) Enrichments of POU5F1, NANOG, SOX2, and KLF4 in the promoter region of POLR3G-dependent transcripts in the genome were examined by comparing the binding of the factors to the proximity (−1,000 from TSS + first exon and intron, if present) of POLR3G-regulated polyA^+^ genes in comparison with all the polyA^+^ genes expressed by hESCs before or after POLR3G knockdown. The overlap of the binding sites of different factors is illustrated in the figure. (B) Binding of POLR3G into the genome of embryonal carcinoma-derived pluripotent stem cells (NT2D1) was studied with ChIP-seq. Binding sites were compared with the transcriptional changes observed in response to POLR3G knockdown and in correlation to ENCODE data available on chromatin status of pluripotent stem cells. The figure shows a snapshot from integrative analysis at the transcriptional start site of *POLG* gene showing genomic binding and transcriptional regulation of the locus by POLR3G.

We next examined the binding of transcription factors, important for pluripotency, in the promoters of the POLR3G-regulated transcripts. For the comparison we used existing chromatin immunoprecipitation sequencing (ChIP-seq) data by Lister et al. (2009) on POU5F1, NANOG, SOX2, and KLF4 binding. Additionally EP300, a histone acetyltransferase and transcriptional co-activator important for stem cell differentiation, was included in the analysis. The promoter was defined as a region including 1,000 bp upstream from the transcription start site (TSS) of the transcript and included first exon and intron, if present. We found significant enrichment for NANOG, SOX2, and KLF4, but not POU5F1, in the promoters of the POLR3G-regulated polyA^+^ transcripts in comparison with similar enrichment in the all the genes expressed in hESCs (Table 3). Interestingly, enrichment of the subunit of Pol III complex, POLR3A, was not observed, although this subunit has been previously reported to co-localize with the pluripotency factors (Alla and Cairns, 2014). A total of 311 (30.5%) of all the POLR3G-dependent polyA^+^ transcripts expressed in hESCs had a binding site for one or more pluripotency factors in the promoter region (Figure 3A).

### POLR3G Binding Sites in Genome

ChIP-seq was carried out to identify the genomic binding sites of POLR3G and direct targets of transcriptional regulation. After several failed experiments with hESC lines, good-quality ChIP-seq data were obtained with one of the two replicates of pluripotent NT2D1 line. Altogether 836 peaks (p = 1.00 × 10^−4^) were detected in the POLR3G ChIP-seq data. First we compared the genomic regions bound by POLR3G with the data available on POLR3A binding sites in H1 hESCs by Alla and Cairns (2014). Of the 330 regions bound by POLR3A, 225 were overlapping with genomic regions bound by POLR3G. These regions were chosen for further analysis to ensure identification of POLR3G-regulated targets with high confidence. The majority of the POLR3G binding sites were localized as clearly defined peaks overlapping with 245 different tRNA genes (Table S5). In addition, 13 peaks were overlapping with non-coding RNAs encoding for components of the ribonucleoproteins (*RMRP*, *RN7SK*, *-7SL1*, *-7SL2*, *RNU6-1*, *-2*, *-8*, *-9*, *-ATAC*, *RNY1*, *-3*, *-4*, *-5*). Two peaks overlapped miRNAs (predicted *AC008738.2*, *MIR3676*) and one vaultRNA (*VTRNA1-3*).

To identify the direct targets of the POLR3G putatively important in the maintenance of hESC status, we identified the overlaps in the genomic binding sites of POLR3G/POLR3A and changes in the transcription of coding or non-coding genes in response to POLR3G knockdown. According to our results, mtDNA polymerase *POLG* was the only protein-coding gene, downregulated (−1.5-fold, FDR = 2.00 × 10^−2^) in response to POLR3G knockdown with POLR3G/POLR3A binding site in the proximal promoter. The exact binding site of POLR3G in *POLG* promoter overlapped with the tRNA-Arg-TCG-1-1 gene, functional genomic element with active or weak promoter histone marks, and numerous transcription factor binding sites based on ENCODE data (ENCODE Project Consortium, 2012) (Figure 3B). Consistently, *tRNA-Arg-CGA* transcript expression was increased on average 5.0-fold (p = 4.00 × 10^−2^) in smallRNA-seq data across the biological replicates. In addition, several other tRNAs bound showed altered expression levels in response to POLR3G knockdown. These included *tRNA-Thr-ACY* (fold change = −4.5, FDR = 3.10 × 10^−2^), *tRNA-Leu-TTA*(*m*) (fold change = −7.15, FDR = 8.00 × 10^−3^), *tRNA-Ala-GCA* (fold change = 3.1, p = 4.90 × 10^−2^) in the smallRNA-seq data, and *tRNA-Gly-GGA* (fold change = −2.2, p = 2.32 × 10^−2^) and *tRNA-Met* (fold change = −2.68, p = 2.10 × 10^−2^) in the mRNA-seq data. Comparison of the transcriptome data and the ChIP-seq data revealed overlaps also in a few other non-coding smallRNAs that had altered expression levels, although the statistical significance was not high. The predicted miRNA *AC008738.2* was bound by POLR3G and expression was decreased 1.6- to 9.3-fold (p = 1.00 × 10^−2^) in response to knockdown. In addition, the smallRNAs *RNY1* (ENSG00000201098.1, fold change = 1.5–5.0, p = 4.60 × 10^−2^), *RNY4* (ENSG00000252316.1, fold change = 1.5–5.9, p = 4.00 × 10^−2^), and *RNY5* (ENSG00000252310.1, fold change = 1.4–2.6, p = 3.40 × 10^−2^), bound by POLR3G, had increased expression in response to POLR3G knockdown in all of the four biological replicates. Also, expression of the VaultRNA (*VTRNA1–3*), bound by POLR3G, had altered expression (1.5- to 1.8-fold) in all of the four biological replicates, although statistical significance was low (p = 2.01 × 10^−1^).

In conclusion, our data indicate that maintenance of stem cell status by POLR3G may be mediated through regulation of *POLG* gene. The regulation of stem cell status may also involve activity of *AC008738.2*, *VTRNA1–3*, a subset of specific tRNA genes, and components of ribonucleoproteins *RNY1*, *RNY4*, and *RNY5*.

## Discussion

POLR3G is a key factor required for maintenance of hESC state, as silencing of POLR3G leads to differentiation and attenuates proliferation of hESCs (Wong et al., 2011, Enver et al., 2005, Haurie et al., 2010). However, the mechanisms by which POLR3G sustains undifferentiated status of hESCs and prevents differentiation have not been characterized in detail. Our results show that POLR3G is required for the maintenance of several types of polyA^+^ and smallRNA transcripts, including protein-coding genes, pseudogenes, lincRNAs, antisense transcripts, and miRNAs in hESCs. Interestingly, the POLR3G-dependent transcriptome is rather specific, as only 5% of the polyA^+^ transcripts and 7% of the smallRNA transcripts in hESCs were POLR3G dependent. This indicates that a large proportion of the transcripts in hESCs are also maintained through POLR3G-independent mechanisms. However, the severe phenotypic and proliferation changes induced in response to POLR3G silencing and accompanied by these transcriptional alterations indicate that POLR3G-dependent regulation of these specific transcripts is crucial for the maintenance of stem cell state and proliferation.

We also found that silencing of POLR3G leads to changes in the expression levels of alternatively spliced transcript variants. Of these we further validated the strongest observation, skipping of exon 9 of the *HDAC7* RefSeq variant 1. Elucidation of the functional significance of this ASE requires further studies. The importance of *HDAC7* in the regulation of early stem cell differentiation and development is supported by a previous study on a mouse stem cell model, which showed that *Hdac7* is required for differentiation of smooth muscle cells and to undergo alternative splicing during differentiation, albeit at different sites affecting usage of exon 1 (Margariti et al., 2009).

The POLR3G-dependent transcripts are functionally linked to embryonic development and key cellular functions important for cellular maintenance and proliferation. The known pluripotency regulators are enriched in the TSSs of these genes, and the majority of the polyA^+^ transcripts are regulated during the differentiation of hESCs to three different germ cell lineages. This regulation of POLR3G-mediated transcripts must be important, enabling lineage specification and further development of the cells and tissues, as previous studies have shown that overexpression of POLR3G in the cells leads to resistance to differentiation (Wong et al., 2011).

The effects of POLR3G knockdown on smallRNA expression were stronger than on polyA^+^ transcriptome, indicating that POLR3G is likely to have an important function in the maintenance of stem cell state and regulation of pluripotency through specific subsets of smallRNAs.

Interestingly, although based on transcriptome analysis, POLR3G is required to maintain expression of multiple types of transcripts in hESCs, only few are direct targets of POLR3G. We found mtDNA polymerase *POLG* to be the only protein-coding gene directly regulated by POLR3G. Therefore, *POLG* is a strong candidate for mediating POLR3G-dependent maintenance of stem cell status. *POLG* encodes the catalytic subunit of the mtDNA polymerase, and is thus required for the proper function and genomic integrity of mitochondria and is essential for early embryonic development. Mitochondrial function has been shown to be crucial for the pluripotency and differentiation of embryonic stem cells (Hance et al., 2005, Facucho-Oliveira et al., 2007, Xu et al., 2013). Silencing of POLG in mouse embryonic stem cells leads to loss of POU5F1 and induction of Brachyury protein expression (Hance et al., 2005, Facucho-Oliveira et al., 2007, Xu et al., 2013), demonstrating the importance of the gene in maintenance of pluripotency.

In addition, binding and POLR3G-dependent expression was detected for a subset of tRNA genes, predicted miRNA *AC008738.2*, *VTRNA1–3*, and ribonucleoproteins *RNY1*, *RNY4*, and *RNY5*, with currently unclear function in the regulation of pluripotency. Strong binding of POLR3G to numerous tRNAs without transcriptional changes in response to knockdown hints at a potential function other than direct regulation of transcription. Further studies are needed to clarify, for example, whether POLR3G has a function in controlling accessibility of chromatin to regulatory factors or mediates regulation of gene transcription through long-range interactions of the chromatin.

In summary, our results provide insights into the molecular mechanisms by which the stem cell-specific subunit of Pol III complex, POLR3G, regulates self-renewal and pluripotency. Furthermore, since Pol III is responsible for transcribing many of the core RNA components of the cytosolic translation machinery, our findings suggest a previously unreported mechanism for coordinated regulation of protein synthesis and mitochondrial biogenesis.

## Experimental Procedures

Full experimental procedures are provided in the Supplemental Information.

### Cell Culture and Differentiation Assays

Human ESC lines were maintained on human foreskin fibroblast feeders or in feeder-free culture conditions on Matrigel (BD Biosciences) in mTeSR1 medium (STEMCELL Technologies) as previously described (Narva et al., 2012, Konki et al., 2016). In feeder-free culture conditions the cells were maintained on Matrigel (BD Biosciences) in mTeSR1 medium (STEMCELL). Differentiation of hESCs was performed as described by Narva et al. (2012). In brief, for spontaneous embryonic body differentiation the cells were plated without feeders and were grown in suspension in standard hESC medium without fibroblast growth factor 2. For retinoic acid-induced differentiation, cells were plated in feeder-free conditions and medium was supplemented with 13.7 μM retinoic acid (Sigma). The karyotypes of the lines were routinely monitored with G-banding and/or KaryoLite BoBs assay (Lund et al., 2012).

### RNA Interference and Transfections of hESC Lines

hESC cultures from two different cell lines (H9p38, HS360p62, HS360p63, HS360p66) were used in POLR3G siRNA experiments as indicated. HS360 was derived in and obtained from the Karolinska Institutet. The cells were first expanded on Matrigel in mTeSR1 for two to three passages to remove feeder cells. Silencing experiments were performed with two different siRNA oligonucleotides (Sigma) and a pool of siRNAs (Santa Cruz Biotechnology) and with non-targeting siRNA control, which were transfected into the cells with Lipofectamine RNAi Max or Lipofectamine 2000 (Invitrogen) according to manufacturer's protocols. The sequence information for the siRNAs can be found in Supplemental Experimental Procedures. Double transfections of siRNAs were performed at 24 and 48 hr after plating of cells on feeder-free conditions. The samples were harvested on day 3 or day 4 after the first transfection for the experiments and analysis. The Cedex XS (Innovatis) system was used to calculate the amount of viable and dead cells in the cultures based on trypan blue staining and cell morphology. From the collected samples total RNA, including smallRNAs, DNA and proteins were extracted simultaneously with a Qiagen Allprep kit.

Silencing of POU5F1, L1TD1, SOX2, and NANOG was carried out as previously described (Narva et al., 2012). In brief, Lipofectamine2000 (Invitrogen) reagent was used for double transfections after 2 or 3 days of plating, and cells were collected for experiments 1–5 days after the second transfection.

### RT-PCR

Taqman real-time qRT-PCR was run as previously described (Lund et al., 2013). RNA was treated with DNase I (Qiagen) during column purification, and a second round of DNase treatment was carried out for 500 ng of total RNA with DNase I Amplification Grade (Invitrogen). To verify that no genomic DNA was present, we performed a negative RT-PCR control measurement with housekeeping gene EF1α from total RNA. cDNA was synthesized using a Superscript II kit (Gibco). The levels of the indicated genes of interest were measured with the 7900HT Fast Real-Time PCR System (Applied Biosystems). The cycles of threshold values (Ct) were compared with those of housekeeping gene to obtain normalized log_2_ expression levels for the transcripts (ΔCt). The primers and probes were designed using a Universal Probe Library Assay Design Center (Roche). Analysis of *HDAC7* variants was carried out with RT^2^ SYBR Green qPCR Mastermix (Qiagen). Primer and probe sequences are listed in Supplemental Experimental Procedures.

### Western Blotting

Protein level analysis was performed as previously described (Narva et al., 2012). In brief, the cells were lysed in buffer with 50 mM Tris-HCl (pH 7.5), 150 mM NaCl, 0.5% Triton X-100, 5% glycerol, 1% SDS, 1 mM Na_3_VO_4_, 10 mM NaF, and 1 mM PMSF. After sonication, protein concentrations were measured with DC Protein Assay (Bio-Rad), after which 6× SDS buffer (0.5 M Tris-HCl [pH 6.8], 28% glycerol, 9% SDS, 5% 2-mercaptoethanol, 0.01% bromophenol blue) was added. After boiling for 5 min, lysates were run in electrophoresis using 10% SDS-PAGE gel and transferred into a nitrocellulose membrane. Membranes were used in western blot analysis with antibodies listed in Supplemental Experimental Procedures.

### Library Preparation and Next-Generation Sequencing

Samples were collected from H9p38 (non-targeted siRNA, siRNA2, and siRNA3), HS360p62 (non-targeted siRNA, siRNA1, and siRNA2), or HS360p66 (non-targeted siRNA and siRNA1) for identification of target genes of POLR3G with next-generation sequencing (NGS) as indicated. The libraries for mRNA-seq with HiSeq2000 platform (Illumina) were prepared from 1 μg (HS360p62 or H9p38) or 0.3 μg (HS360p66) of total RNA. The sample preparation was performed with TrueSeq RNA Sample Preparation Kit v3 (Illumina) according to the kit manual. The smallRNA library preparation for the HS360p66 sample was unsuccessful, most likely due to the decreased presence of smallRNAs in the total RNA pool. The libraries for smallRNA-seq were prepared from 1 μg of total RNA, containing the smallRNA fraction, with Illumina TruSeq smallRNA Sample Preparation kit according to the manual. The libraries were size selected to enrich the smallRNAs with size less than 250 bp. The cluster generation was performed automatically with a c-Bot instrument (Illumina). The mRNA-seq libraries were sequenced with 2 × 50 bp and smallRNA-seq with 1 × 50 bp chemistry and HiSeq2000 platform (Illumina).

### Data Analysis of the mRNA-Seq Data

The sequence data were aligned against hg19 reference genome assembly and annotated with the gencode (v19) gene annotations (concatenated with tRNA annotations from the UCSC genome browser) using TopHat2 (Trapnell et al., 2009). RPKM values were computed using the total exon length and, with genes that have multiple transcripts, the total exon length corresponding to the transcript with longest total exon length was used. Genes were filtered using a minimum of 0.1 RPKM value in at least two replicates in at least one condition to exclude genes with expression levels that are too low to differentiate from noise (Ramskold et al., 2009). An additional filter was applied to extract transcripts with a minimum RPKM value of 0.5 in all of the biological replicates in one of the conditions. EdgeR (McCarthy et al., 2012), a tool that handles multifactor experimental designs, was used to determine differential expression to account for the paired design of the input data. Adjusted p values were computed with the Benjamini-Hochberg method and an FDR of 0.05 was used as a primary threshold for significance unless otherwise stated. Minimum fold change cutoff of 1.5 was applied to extract the genes showing consistent differences between the sample groups.

### Data Analysis of the SmallRNA-Seq Data

SmallRNA-seq data were trimmed and mapped to the miRBase database (build hg19) with Bowtie2 and to the hg19 human reference genome (with gencode v19 and tRNA annotations) with TopHat2. To minimize background noise, we filtered smallRNA transcripts by requiring a minimum RPM of 0.5 in all of the biological replicates in one of the conditions. EdgeR was used to identify the differentially expressed smallRNAs and a minimum expression change of 1.5-fold was used. In addition, an adjusted p value cutoff (0.05) was applied to extract the statistically significant differences.

### Functional Enrichment Analysis

Ingenuity Pathway Analysis Tool (Qiagen, www.ingenuity.com) was used to examine functional enrichments of the POLR3G-dependent transcripts. This tool calculates the likelihood for enrichment of the examined molecules in each functional category with right-tailed Fisher exact test as indicated in the results.

### Comparison with Publicly Available Datasets

POLR3G levels in pluripotent stem cells and other cell types were extracted and visualized from the data available from ESTOOLS data@hand database (Kong et al., 2013). The regulation of the POLR3G-dependent genes during differentiation was examined in the data available from Gifford et al. (2013). The publicly available ChIP-seq data were utilized to examine the binding of known pluripotency factors and POLR3A to the proximity of POLR3G-dependent genes (Lister et al., 2009, Alla and Cairns, 2014). To extract the binding sites that overlap with promoter regions of polyA^+^ transcripts expressed in hESCs or regulated in POLR3G manner, we first converted the genomic coordinates of the binding sites from the hg18 assembly to hg19 using liftOver (http://genome.ucsc.edu/). A promoter region was defined as 1,000 bp upstream of the TSS up to the end of the first exon or the first intron (if present). In cases of multiple transcript IDs for one gene ID, one transcript ID whose gene type is the same as its transcript type was randomly selected. The number of POLR3G-dependent genes whose promoter overlapped at least one binding site was counted and the statistical significance of this overlap was obtained using the hypergeometric test (wherein the population size was the total number of expressed genes in hESCs, the sample size was the number of POLR3G-dependent genes, and the number of successes in the population was the number of expressed genes overlapping with a binding sites). The filtering criteria used for extracting the POLR3G dependent genes for the enrichment analysis were absolute average fold change ≥1.5 (consistent in direction of fold change across paired samples) and FDR (Benjamini-Hochberg) ≤0.05. Because of their multiple genomic locations (based on the annotations used in this analysis), tRNAs were excluded from this analysis.

### Splicing Analysis

Splicing analysis was performed using rMATS (Shen et al., 2014), which uses a hierarchical model to estimate the proportion of ASEs based on the binomial distribution to model read counts and the bivariate normal distribution to model variance between replicates and to account for the covariance between paired case and control samples. The following analysis parameters were used: anchor length ≥8 (default) and the absolute difference in proportion of splice variants ΔΨ >0.2. The transcript annotations were from gencode v19. Significant ASEs (FDR < 0.05) using junction counts and counts from both junction and non-junction reads were combined. MISO (Katz et al., 2010), a Bayesian method for detecting differential splicing, was used, requiring number of reads mapping to splicing event ≥20. ASEs were identified separately for the three pair replicates and filtered with the following parameters: Bayes factor ≥5, ΔΨ >0.2, and number of reads supporting inclusion/exclusion isoform in case/control samples ≥10. Ensembl splicing event annotations were used (Aken et al., 2016).

### POLR3G ChIP-Seq

The detailed protocol is available in Supplemental Experimental Procedures. In brief, NT2D1 cells (passages 34 and 35) were fixed with 1% formaldehyde (#28906, Thermo Fisher Scientific) for 10 min and the chromatin was isolated. The chromatin was fragmented by sonication for 5 min with Bioruptor Pico (Diagenode). ChIP was carried out with antibody raised against human POLR3G (Renaud et al., 2014) (SZ3070, a gift from Pascal Cousin and Professor Nouria Hernandez, University of Lausanne), and H3K4me3 (C15410003, Diagenode) was used as a positive control. The quality and quantity of the ChIP-DNA was determined with Fragment Analyzer (Kem-en-tec) and Qubit (Thermo Fisher Scientific). The libraries were prepared with Microplex Library Preparation kit v2 (Diagenode) and NGS was carried out with Illumina HiSeq3000 with 1 × 50 bp chemistry. The data were analyzed in the Galaxy (usegalaxy.org) (Afgan et al., 2016). The quality of the sequencing data was analyzed with FastQC (Andrews, 2016). A total of 14,843,348–27,632,141 raw reads per sample were obtained. The duplicate rates were 13%–27%. The reads were filtered with Trimmomatic 0.32 (Bolger et al., 2014) to remove low-quality reads; reads with average quality below 20 were removed and reads with length 20–50 bp were retained. The reads were mapped to hg19 genome with Bowtie2 2.2.6 (Langmead et al., 2009, Langmead and Salzberg, 2012) and non-unique reads were discarded. The number of unique aligned reads was 10,424,601–20,684,726 per sample. The ChIP peaks were called with MACS 1.0.1 (Zhang et al., 2008) with default parameters, except an arbitrary shift size of 100 bp was used instead of shifting model, and p value cut off was 1.00 × 10^−4^. The peaks were visualized in IGV 2.3.72 (Robinson et al., 2011, Thorvaldsdottir et al., 2013). The overlapping peaks in the datasets were identified with BEDTools (Quinlan and Hall, 2010).

## Author Contributions

R.J.L. had the main responsibility in the coordination of the work, culture of the cells for knockdown experiments, knockdowns, characterization of the cell, sampling, analysis of ChIP-seq data, in-depth analysis and interpretation of the transcriptome data and other results, and preparation of the final version of the manuscript and figures. N.R. participated in the manuscript preparation, was responsible for the qRT-PCR validations, and together with E.N. and M.R.E. carried out the differentiation series and western blot analysis (Figure S1A). M.M. had the main responsibility in the processing and bioinformatics analysis of Illumina NGS data. L.K. prepared the NGS libraries with the assistance of FMSC staff. M.R.E. carried out the experiments in Figure S1C. V.K. and M.N. analyzed Helicos single-molecule sequencing RNA-seq and ChIP-seq data, which were not included in the final version of the manuscript due to quality issues in the ChIP-seq data. O.H. and H.S. provided the HS360 cell line for the study, helped to establish the hESC cultures in our laboratory, and participated in the early phase of the study, which helped us to detect enrichment of POLR3G hESCs. E.K. participated in the interpretation of the results on POLG finding. A.L. helped to implement the Helicos and Illumina NGS data-processing pipelines in our laboratory and participated in the early phase of the data analysis. H.L. supervised the bioinformatics analysis of the Illumina NGS data. With the assistance of O.R. and RJ.L., R.L. supervised the work. All authors contributed to the final interpretation of the results and critical review of the manuscript.

## Figures and Tables

**Table 1:** Functional Enrichment of the POLR3G-Dependent PolyA^+^ Transcripts

Functional Category	No. of Molecules	Fisher Exact Test p Value
Cellular and Molecular Functions		

Cellular assembly and organization	174	0.0025
Cellular function and maintenance	150	0.0024
Cell cycle	109	0.0026
DNA replication, recombination, and repair	41	0.0023
Cell morphology	148	0.0024

Physiological System Development and Function		

Embryonic development	122	0.0026
Nervous system development and function	145	0.0024
Organ development	79	0.0021
Organismal development	172	0.0023
Tissue development	139	0.0024

**Table 2:** Alternative Splicing Events Detected by MISO Statistical Model in Response to Silencing of POLR3G in hESCs

Splicing Event (Genomic Loci)	Category	Ensembl ID	Gene ID	Bayes Factor	Δψ
chr12:48189990:48190081:- @chr12:48189689:48189799:- @chr12:48189370:48189550:-	SE	ENSG00000061273	HDAC7	≥1.00E+12	≥0.21
chr17:74087224:74087316:- @chr17:74086410:74086478:- @chr17:74085256:74085401:-	SE	ENSG00000182473	EXOC7	≥76.48	≤−0.20
chr19:2226182:2227126:- @chr19:2227736:2228381:- @chr19:2229784:2232577:-	SE	ENSG00000104885	DOT1L	≥100E+12	≤−0.24
chr15:137356720:137356886:- @chr15:137354644:137354835:- @chr15:137353991:137354203:-	SE	ENSG00000031003	FAM13B	≥6.95	≤−0.27
chr4:119459016:119459112I 119459163:+ @chr4:119461374:119461544:+	A5SS	ENSG00000154608	CEP170P1	≥11.89	≤−0.3
chr7:23562051-23561740:- @ chr7:23561459-23561326:-	RI	ENSG00000164548	TRA2A	≥610.64	≤−0.24

See also Figure S3.

**Table 3:** Binding and Enrichment of Regulatory Factors in the Promoters of POLR3G-Dependent PolyA^+^ Genes in Comparison with All Detected Genes

DNA Binding Factor	No. of Bound Genes in the Genome	No. of Bound Genes in hESCs	No. of Bound POLR3G-Dependent Genes	Hypergeometric Test p Value	Adjusted p Value (Benjamini-Hochberg)
NANOG	25,071	1,668 (12.2%)	166 (16.3%)	3.64 × 10^−5^	2.19 × 10^−4^
KLF4	3,793	1,116 (8.2%)	105 (10.3%)	4.55 × 10^−4^	1.36 × 10^−3^
SOX2	5,682	467 (3.4%)	49 (4.8%)	6.57 × 10^−3^	1.31 × 10^−2^
EP300	3,093	280 (2.1%)	37 (3.6%)	9.08 × 10^−3^	1.36 × 10^−2^
POU5F1	3,889	1,524 (11.1%)	123 (12.1%)	1.74 × 10^−1^	2.09 × 10^−1^
POLR3A	389	40 (0.3%)	3 (0.3%)	5.81 × 10^−1^	5.81 × 10^−1^

## Accession Numbers

The NGS data are available at the NCBI GEO database with series accession number GEO: GSE94696 (Edgar et al., 2002).

## Supplemental Information

Document S1. Supplemental Experimental Procedures and Figures S1–S3

Table S1. Human ESC Reference Transcriptome

Table S2. POLR3G-Dependent Transcriptome in hESCs

Table S3. POLR3G-Dependent Genes Up- or Downregulated during Endoderm, Ectoderm, and/or Mesoderm Differentiation, Related to Results

Table S4. POLR3G-Dependent Splicing Events in hESCs

Table S5. Overlapping Genomic Regions Bound by POLR3G in NT2D1 Cells and POLR3A in H1 Cells, Related to Figure 3

Document S2. Article plus Supplemental Information
