# Supplementary material for: RNA Polymerase III Subunit POLR3G Regulates Specific Subsets of PolyA+ and SmallRNA Transcriptomes and Splicing in Human Pluripotent Stem Cells
Source: Stem Cell Reports. 2017 May 9;8(5):1442–54. doi: 10.1016/j.stemcr.2017.04.016 (PMC5425787; doi:10.1016/j.stemcr.2017.04.016)
Supplement: Document S1. Supplemental Experimental Procedures and Figures S1–S3 [file mmc1.pdf]

Stem Cell Reports, Volume *8*

Supplemental Information

RNA Polymerase III Subunit POLR3G Regulates Specific Subsets of PolyA$^+$ and SmallRNA Transcriptomes and Splicing in Human Pluripotent Stem Cells

Riikka J. Lund, Nelly Rahkonen, Maia Malonzo, Leni Kauko, Maheswara Reddy Emani, Virpi Kivinen, Elisa Närvä, Esko Kemppainen, Asta Laiho, Heli Skottman, Outi Hovatta, Omid Rasool, Matti Nykter, Harri Lähdesmäki, and Riitta Lahesmaa

Figure S1. Related to Figure 2A.

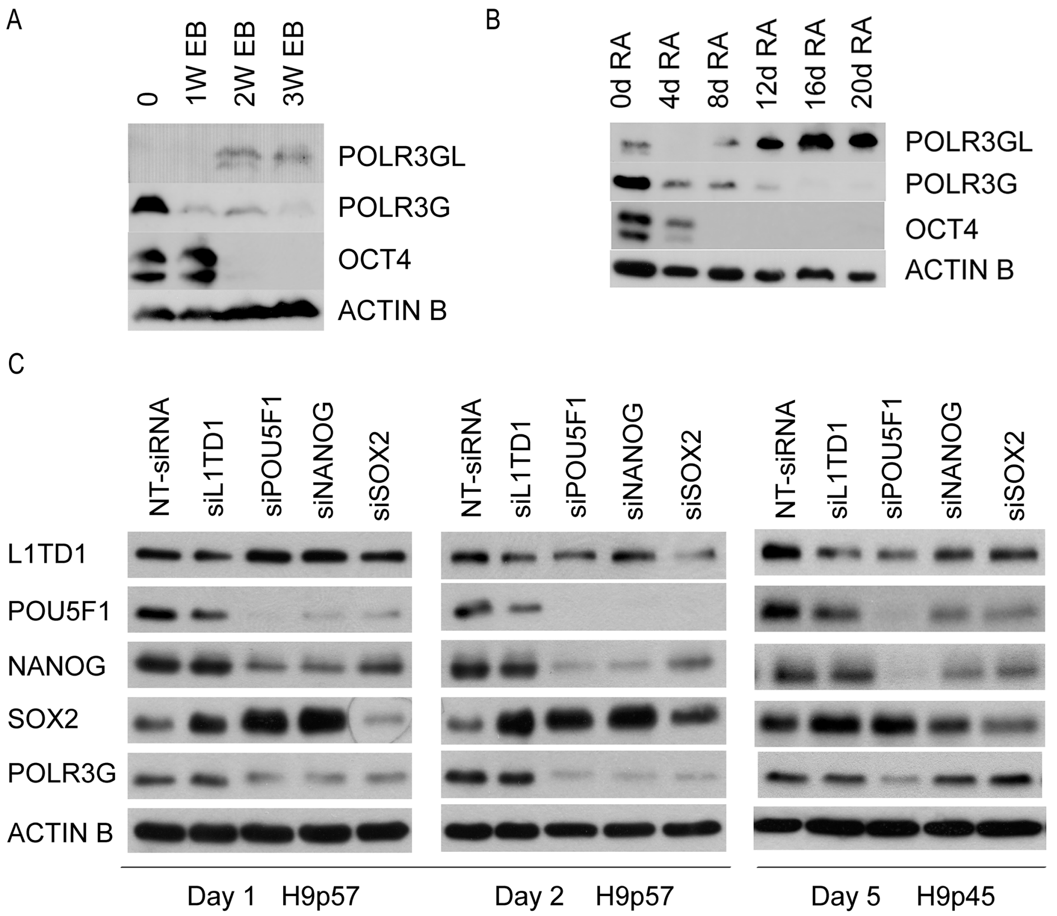

Figure S1. POLR3G is rapidly downregulated during human embryonic stem cell differentiation and is expressed in POU5F1 dependent manner. Human embryonic stem cells were exposed to retinoic acid (RA) or were allowed to differentiate spontaneously to embryonic bodies (EB) in hESC medium without FGF2 and feeders. Samples were collected at indicated time points and were analysed with Western blot to examine levels of POLR3G during differentiation. Actin B was used as a loading control, POU5F1 (OCT4) as a differentiation control. In addition, an alternative variant POLR3GL was measured in parallel. In the figure **A**) is representative data for protein level changes of POLR3G, POLR3GL and stem cell marker POU5F1 in response to spontaneous EB differentiation of H9 line. In the figure **B)** is the representative data for western blot analysis of POLR3G and POLR3G expression in response to RA induced differentiation of H9 line. **C)** H9 cells were transfected with siRNAs targeting stem cell markers L1TD1, POU5F1, NANOG, and SOX2. Samples were collected from three different biological replicates after 1 and 2 days (p57), 3 days (p45, p54 and p57) and 5 days (p45) of transfection. Western blot analysis was carried out to determine knockdown efficiency and effect on POLR3G protein levels. In the entire set of 6 samples POU5F1 knockdown led to decreased protein expression of POLR3G. The effect of other knockdowns was variable. In the figure is the representative data from three sample series. NT = non-targeted siRNA.

Figure S2. Related to Figure 2B-E.

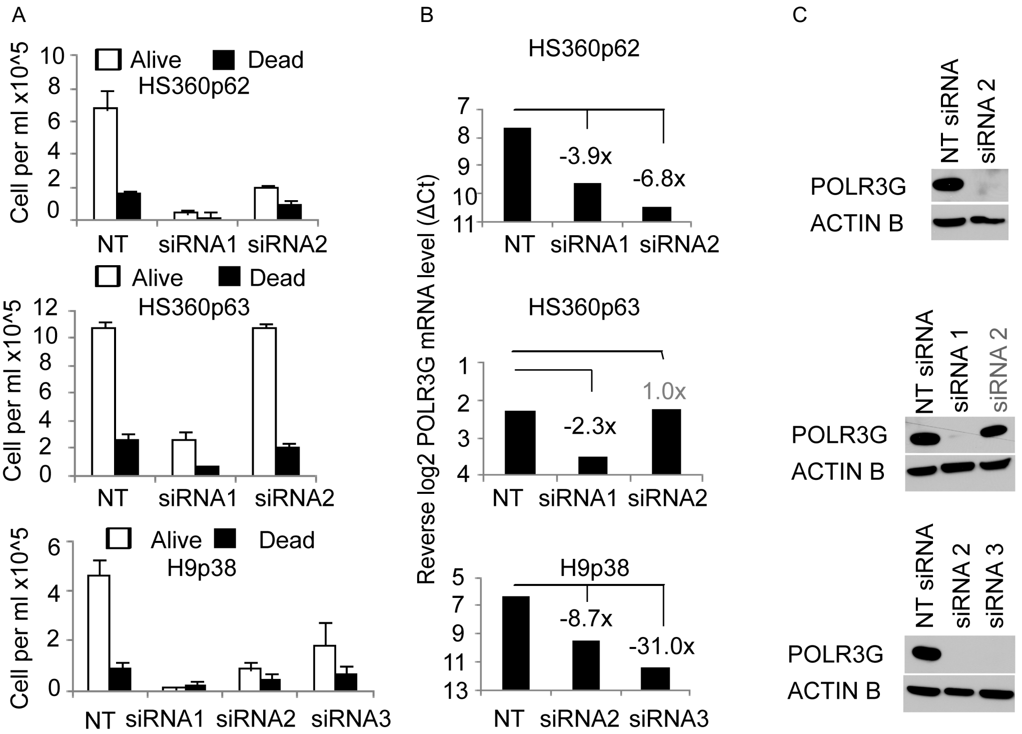

Figure S2. POLR3G is required for hESC self-renewal and proliferation. Two different human ESC lines, H9 and HS360, were transfected at indicated passages (HS360p62, HS360p63, H9p38) with two different siRNAs (siRNA1, siRNA2) and with a siRNA pool (siRNA3) targeting POLR3G. The samples were collected and analyzed after 3 days (HS360) or 4 days of second transfection (H9p38). The error bars for standard deviation indicate variation in replicated cell counts for samples collected from three individual cell culture wells for each sample type as indicated in the figure. **A**) Silencing of POLR3G led to strong decrease in cells numbers as revealed by cell analysis with Cedex XS system (innovates, Roche Applied Science). No effect on viability was observed based on Trypan Blue staining. **B**) When enough material was available after silencing of POLR3G the knockdown efficiency was confirmed at mRNA level with quantitative real-time RT-PCR **C**) and at protein level with western blot analysis. NT = non-targeted siRNA. Rep # = replicate number. HS360p63 siRNA2 is marked as grey, as knockdown was not significant. Representative data from three different experiments, with two cell lines and with two or more different siRNAs is shown for each replicate in the figure. The error bars represent technical replicates from at least three measurements.

Figure S3. Related to Table 3.

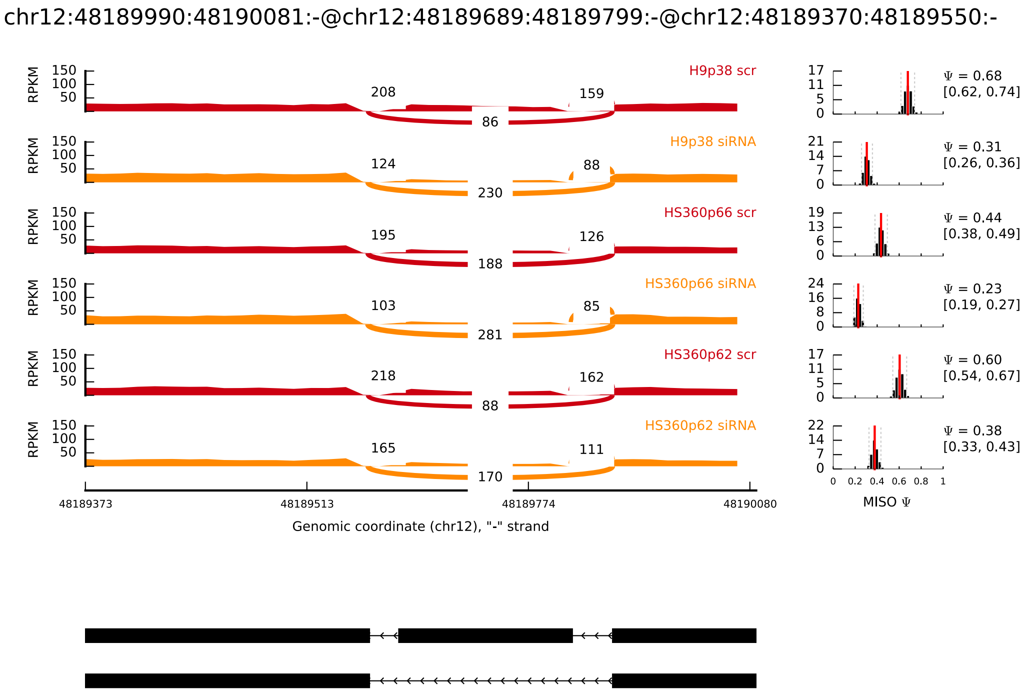

Figure S3. Skipping of exon 9 in HDAC7 transcript variant 1 in response to POLR3G silencing. Alternative splicing was compared with MISO statistical method in human ESCs treated with non-targeted siRNA (NT-siRNA or scr) and siRNAs targeting POLR3G in three independent biological replicates as indicated in the figure. The figure illustrates the alternative splicing event, skipping of exon in HDAC7, which was found to be statistically significant by both MISO (Table 3) and rMATS (Table S4) methods. The estimates of isoform expression (Ψ values) and confidence intervals (Bayes factors) are displayed in the right panel.

Supplemental Experimental Procedures

The siRNA oligos used in the study

The knockdown of POLR3G was carried out using two different siRNA siRNA1: CCAGUACCACUGAAAACAGdTdT [S1], siRNA2: UGACGAUGAUGCCGCAGAA [S2] and a commercial siRNA pool siRNA3: pool of 3 sequences (sc-43507, Santa Cruz Biotechnology). POLR3G (OCT4) was silenced with siRNA sequence AAGGAUGUGGUCCGAGUGUGG [S3], NANOG with AAGGGUUAAGCUGUAACAUAC [S3] and L1TD1 with GCAAGGACGTATCAGCAATTA [S3]. Non-targeting siRNA was used as a control: CCUACAUCCCGAUCGAUGAUG [S4].

Quantitative Real-Time RT-PCR oligos

Target	Sequence
POLR3G	5'-CGCAGAACAGGAGGAATATGA-3'
	5'-CACTGTCTGCGCCAAAATC-3'
	Probe 35 (Roche probe library)
POLR3GL	5'- GCCCAGTACATTTCAAGTTGG -3'
	5'- GGGCCTGGGTATTCAGAGAT -3'
	Probe 62 (Roche probe library)
HDAC7_1	5'-GGCTCGGAGGCGCTCTT-3'
	5'-AAGGACACTGTCGGCAAGG-3'
HDAC7_2	5'-CTCCCCAAGTAGTAGCAGCAC-3'
	5'-CACTGTCAGCCTCCGAGC-3'
EF1α	5´-CTGAACCATCCAGGCCAAAT-3´
	5´-GCCGTGTGGCAATCCAAT-3´
	Probe: 5´-(FAM)-AGCGCCGGCTATGCCCCTG-(TAMRA)-3´
Actin B	5'- CCAACCGCGAGAAGATGA -3'
	5'- CCAGAGGCGTACAGGGATAG -3'
	Probe: 64 (Roche probe library).

Antibodies used in the Western blotting

Antibody	Manufacturer	Dilution	Catalog number
POLR3G	Santa Cruz Biotechnology	1:500	sc-21754
POLR3GL	Sigma	1:1000	HPA027288
POU5F1	Santa Cruz Biotechnology	1:500	sc-9081
NANOG	R&D Systems	1:1000	AF1997
SOX2	R&D Systems	1:1000	AF2018
L1TD1	Sigma	1:5000	HPA028501
β-actin	Sigma	1:10 000	A5441
GAPDH	HyTest	1:10 000	5G4
Anti-Rabbit-HRP	BD Pharmingen	1:10 000	554021
Anti-Mouse-HRP	Santa Cruz Biotechnology	1:10 000	sc-2005
Anti-Goat-HRP	Santa Cruz Biotechnology	1:10 000	sc-2020.

Nuclei isolation and Chromatin Immunoprecipitation

NT2D1 (NTERA2 clone D1) cells were culture in Dulbeccos's Modified Eagle's Medium (Sigma) containing 15 % of FBS (#S18190-500, Biowest) and 200 mM L-glutamine (Biowest). At passages 37 (replicate 1) and 39 (replicate 2) the cells were fixed with 1 % formaldehyde (#28906, ThermoFisher Scientific) for 10 min in a buffer containing 50 mM Hepes-KOH, pH 7.3, 100 mM NaCl, 1 mM EDTA, 0.5 mM EGTA. The fixing reaction was stopped with 125 mM glycine at RT for 5 min. The glycine solution was removed and the cells were washed twice with ice cold 1x PBS and collected into a conical tube in 5 mls of ice cold 1xPBS by scraping. The cells were pelleted.

The nuclei were isolated by lyzing the cells in buffer I (50 mM Hepes-KOH, pH 7.5, 140 mM NaCl, 1 mM EDTA, 10 % glycerol, 0.5 %NP-40, 0.25 % Triton X-100) for 5 min on ice. The samples were pelleted (2 000 g, 5 min, 4°C) and resuspended in buffer II (10 mM Tris-HCl, pH 8.0, 200 mM

NaCl, 1 mM EDTA, pH 8.0, 0.5 mM EGTA, pH 8.0) with protease inhibitors (#11697498001, Roche). After 5 min incubation on ice the samples were pelleted and resuspended in buffer III (10 mM Tris-HCl, pH 8.0, 200 mM NaCl, 1 mM EDTA pH 8.0, 0.5 mM EGTA pH 8.0, 0.1 % Na-Deoxycholate, Sigma, D6750, 0.5 % N-lauroylsarcosinecontaining, protease inhibitors). After 5 min incubation on ice the chromatin was pelleted and washed again with buffer III. The chromatin was resuspended in buffer III (~50 000 cells/100 µl) and sonication was carried out with Bioruptor Pico, 30 s on, 30 s off for 5 min.

The chromatin immunoprecipitation (ChIP) was carried out with antibody raised against human POLR3G (SZ3070, a generous gift from Pascal Cousin and Professor Nouria Hernandez, University of Lausanne, Switerland) [S5]. In addition, antibody against H3K4me3 (C15410003, Diagenode) was used as a positive control.

The magnetic beads (#11203D, Invitrogen) were vortexed. For each ChIP reaction 11 µL of beads were aliquoted and incubated on magnet for 2 min. The supernatant was removed. The beads were washed three times with PBS containing 0.5 % BSA. For each ChIP reaction 0.5 µg of antibody was added into 500 µL of PBS+0.5% BSA solution. The antibodies were attached into the beads by incubation for 4 - 8 hours at 4°C in rotation.

The tubes were filled with 500 µl of buffer III + 1 % Triton X-100 + protease inhibitors. An aliquot of the sonicated chromatin was saved as an input control. The supernatant was removed from the beads. An aliquot of 500 µl of sonicated chromatin was added for each ChIP reaction, into the tubes with antibody-bead complexes, and the samples were incubated over night at 4°C in rotation.

The chromatin-antibody-bead complexes were collected and washed with RIPA buffer (50 mM Hepes pH 8.0, 1 % NP-40, 0.7 % DOC, 0.5 M LiCl, 1 mM EDTA, protease inhibitors) for five times and once with TE buffer. The beads were resuspended in 100 µL of elution buffer (10 mM Tris pH 8.0, 1 mM EDTA, 1 % SDS). The immunoprecipitates were incubated at 65 °C for 5 h in a thermomixer with 1 400 rpm shaking. The supernatants were collected and 1 ul of Proteinase K (20 mg/ml, #EO0491, ThermoFisher Scientific) was added into the samples. The samples were incubated at 55 °C for 1 h. The ChIP-DNAs were purified with MinElute PCR Purification kit.

Supplemental References

S1. Enver, T. et al. Cellular differentiation hierarchies in normal and culture-adapted human embryonic stem cells. Hum. Mol. Genet. 14, 3129-3140 (2005).

S2. Haurie, V. et al. Two isoforms of human RNA polymerase III with specific functions in cell growth and transformation. Proc. Natl. Acad. Sci. U. S. A. 107, 4176-4181 (2010).

S3. Narva E. et al. RNA-binding protein L1TD1 interacts with LIN28 via RNA and is required for human embryonic stem cell self-renewal and cancer cell proliferation. Stem Cells 30, 452-460 (2015).

S4. Berra, E. et al. HIF prolyl-hydroxylase 2 is the key oxygen sensor setting low steady-state levels of HIF-1alpha in normoxia. EMBO J. 22, 4082-4090 (2003).

S5. Renaud, M. et al. Gene duplication and neofunctionalization: POLR3G and POLR3GL. Genome Res. 24, 37-51 (2014).
